# Ultrasonography for clinical decision-making and intervention in airway management: from the mouth to the lungs and pleurae

**DOI:** 10.1007/s13244-014-0309-5

**Published:** 2014-02-12

**Authors:** Michael S. Kristensen, Wendy H. Teoh, Ole Graumann, Christian B. Laursen

**Affiliations:** 1 Department of Anaesthesia, Rigshospitalet, University Hospital of Copenhagen, Blegdamsvej 9, 2100 Denmark; 2 Department of Women’s Anaesthesia, KK Women’s & Children’s Hospital Singapore, Duke-NUS Graduate Medical School, 100 Bukit Timah Road, Singapore, 229899 Singapore; 3 Department of Radiology, University of Southern Denmark, Hospital Littlebelt, Kabbeltoft 25, 7100 Vejle, Denmark; 4 Research Unit at the Department of Respiratory Medicine, Odense University Hospital, Sdr. Boulevard 29, 5000 Odense C, Denmark

**Keywords:** Airway management, Intubation, Intratracheal, Tracheostomy, Pneumothorax, Ultrasonography

## Abstract

**Objectives:**

To create a state-of-the-art overview of the new and expanding role of ultrasonography in clinical decision-making, intervention and management of the upper and lower airways, that is clinically relevant, up-to-date and practically useful for clinicians.

**Methods:**

This is a narrative review combined with a structured Medline literature search.

**Results:**

Ultrasonography can be utilised to predict airway difficulty during induction of anaesthesia, evaluate if the stomach is empty or possesses gastric content that poses an aspiration risk, localise the essential cricothyroid membrane prior to difficult airway management, perform nerve blocks for awake intubation, confirm tracheal or oesophageal intubation and facilitate localisation of tracheal rings for tracheostomy. Ultrasonography is an excellent diagnostic tool in intraoperative and emergency diagnosis of pneumothorax. It also enables diagnosis and treatment of interstitial syndrome, lung consolidation, atelectasis, pleural effusion and differentiates causes of acute breathlessness during pregnancy. Patient safety can be enhanced by performing procedures under ultrasound guidance, e.g. thoracocentesis, vascular line access and help guide timing of removal of chest tubes by quantification of residual pneumothorax size.

**Conclusions:**

Ultrasonography used in conjunction with hands-on management of the upper and lower airways has multiple advantages. There is a rapidly growing body of evidence showing its benefits.

**Teaching Points:**

• *Ultrasonography is becoming essential in management of the upper and lower airways.*

• *The tracheal structures can be identified by ultrasonography, even when unidentifiable by palpation.*

• *Ultrasonography is the primary diagnostic approach in suspicion of intraoperative pneumothorax.*

• *Point-of-care ultrasonography of the airways has a steep learning curve.*

• *Lung ultrasonography allows treatment of interstitial syndrome, consolidation, atelectasis and effusion.*

## Introduction

Management of the upper and lower airways and diagnosis of pathological conditions and complications are essential clinical skills for any physician in anaesthesia, emergency medicine, respiratory medicine and intensive care settings. As inadequate airway management remains a major contributor to patient mortality and morbidity [1], any clinical tool that can improve airway management must be considered as an adjunct to the conventional clinical assessment. Ultrasonography (US) has many obvious advantages (it is safe, quick, repeatable, portable, widely available and gives real-time dynamic images) in the emergency setting and several studies have evaluated its role in both management of the airways and diagnosis of pathology in the upper and lower airways. The purpose of this review is to give an overview of how to obtain bedside real-time ultrasonography of the upper and lower airway and of other organs crucial for airway management, and how to use this in clinical practice in improving the management of the airway. Ultrasonography must be used dynamically—in direct conjunction with both airway procedures and assessment of a patient with suspected pathology in the airways—for maximum benefit in airway diagnostics and management.

## Materials and methods

This is a narrative review combined with a structured Medline literature search. The following search terms were used:

1. (ultrasound OR ultrasonic OR ultrasonography OR ultrasonographically OR sonography OR ultrasonographic) AND (vocal cords OR vocal folds OR subglottic OR epiglottis OR extubation OR cricothyrotomy OR tracheostomy OR airway OR larynx OR laryngeal OR laryngoscopy OR endotracheal tube OR endotracheal intubation OR tracheal intubation OR oesophageal intubation OR laryngeal mask airway OR ventilation OR nasogastric OR gastric tube)
2. (ultrasound OR ultrasonic OR ultrasonography OR ultrasonographically OR sonography OR ultrasonographic) AND (lung OR pneumothorax OR effusion OR empyema OR lung consolidation OR pneumonia OR pulmonary embolism OR atelectasis OR tumor OR malignancy OR pulmonary oedema OR heart failure OR lung fibrosis OR interstitial lung disease OR asthma OR chronic obstructive pulmonary disease OR dyspnoea OR extended Focused assessment with sonography for trauma)

The reference list of the retrieved articles were additionally scrutinised for relevance.

## Results and discussion

### Ultrasonography and air

From a historical radiological point of view, air and bony structures have been considered enemies of ultrasonography. Air from bowel gas is a well-known challenge in ultrasonography, e.g. the air reduces the diagnostic view to underlying abdominal parenchyma. Several studies have, however, established that the air artefacts can often be used in clinical practice, rather than being an annoyance to the physician performing sonography [2]. By understanding the generated air artefacts seen with ultrasonography, the information can serve as an important diagnostic tool. The technical explanation of air artefacts and how to understand the information gleaned from them are explained below.

Due to differences in velocity and acoustic impedance of ultrasound between normal tissue and air-filled parenchyma (lung, trachea, etc.), a total reflection of ultrasound occurs. Air has a high attenuation coefficient for the transmission of ultrasound. Therefore normal lung parenchyma appears as a homogenous grey picture on B-mode and often, special reverberation artefact creates multiple parallel white lines on the screen. Furthermore the presence of characteristic artefacts can be used as an indirect marker of lung disease. The most useful of these artefacts are the B-line artefacts, which are believed to occur when the density of the lung is increased, for example in interstitial oedema or pulmonary fibrosis [2–6]. The ultrasonic waves are believed to cause resonance in the lung interstitium with increased density: this continuing echo-signal appears on the screen as a strong hyperechoic, laser-like, vertical line from the pleura and extends to the bottom of the field of view, moving synchronously with lung sliding [2–6].

#### Transducer selection

The linear medium to high frequency (5–14 MHz) transducer is suitable for imaging superficial airway structures (within 0–5 cm beneath the skin surface). The curved low-frequency transducer (∼4.0 MHz) is most suitable for obtaining sagittal and parasagittal views of the tongue and structures in the submandibular and supraglottic regions, mainly because of its wider field of view. Linear transducers, which are used for assessment of the upper airways, provide excellent images of superficial structures such as ribs and the pleura, but deeper structures can be difficult to assess. A microconvex transducer (∼8.0 MHz) is a good all-round transducer for focused ultrasonographic examination of the lungs, since most microconvex transducers have an acceptable image quality of both superficial (pleura) and deeper structures (e.g. lung consolidation, atelectasis). Furthermore, microconvex transducers are often small, which makes is easier to access the posterior thoracic wall when the patient can only be examined in the supine position [7]. An alternative to the microconvex transducer for examination of the lungs is the curved low-frequency transducer (∼4.0 MHz), which also has an acceptable image quality of both superficial and deeper structures [6, 8]. Since visualisation of superficial and deep structures is needed, it is important to continuously optimise transducer frequency in order to obtain the best possible images. The presence or absence of artefacts such as B-lines are an important part of lung ultrasound (LUS), hence one should be mindful to deactivate any image optimisation software that is inherently built-in to newer ultrasound machines, as this would remove or diminish the presence of these useful artefacts when performing LUS.

### Ultrasonographic visualisation of structures relevant to airway management

**Fig. 1:**
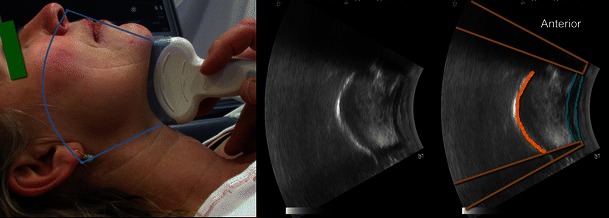
Mouth and tongue. *Left*: The curved, low frequency transducer and the area covered by the scanning (*light blue*). *Middle*: The resulting ultrasound image. *Right*: The dorsal surface of the tongue (*orange*), the muscles in the floor of the mouth (*turquoise*). The *shadows* (*brown*) from the mentum of the mandible anteriorly and from the hyoid bone posteriorly

**Fig. 2:**
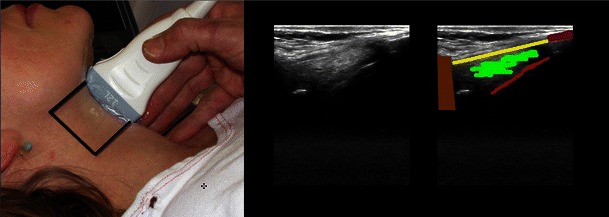
Midline sagittal scan from the hyoid bone to the proximal part of the thyroid cartilage. *Left*: The *black outline* shows the area covered by the scanning. *Middle*: The scanning image. *Right*: The shadow from the hyoid bone (*brown*); the thyro-hyoid membrane (*yellow*); posterior surface of part of epiglottis (*red*); pre-epiglottic fat (*green*); thyroid cartilage (*purple*)

**Fig. 3:**
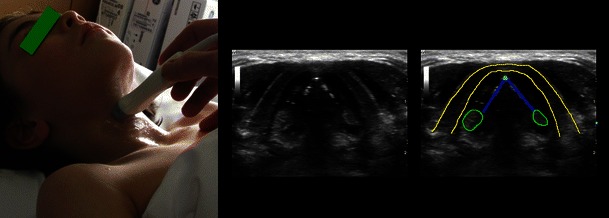
Larynx and vocal cords. *Left*: Transverse midline scan over the thyroid cartilage (in an 8-year-old boy). Vocal cords (*dark blue*); anterior commisure (*light blue*); arytenoid cartilages (*green*); thyroid cartilage (*yellow*)

The airway related structures that can be visualised by computed tomography (CT) can also reliably be identified by US [9]. We have previously described how to visualise the airway from *the tip of the chin to the mid-trachea* with ultrasonography in easy-to-follow steps [10]. The following structures that are relevant to airway management can be examined with US: the *mouth and tongue* [11–17] (Fig. 1), *oro-pharynx* [18] [19], *hypo-pharynx* [20], *hyoid Bone* [21], *epiglottis* (Fig. 2) and *larynx*—due to the superficial location of the larynx, US offers images of higher resolution than CT or magnetic resonance imaging (MRI) when examined with a linear high-frequency transducer [22]. The thyroid and the cricoid cartilages show variable but progressive calcification with advancing age throughout life, whereas the epiglottis stays hypoechoic [23–26]. *The vocal cords* (Fig. 3) appear hypoechoic but are medially outlined by the hyperechoic vocal ligaments [24, 27]. The false vocal cords lay parallel and cephalad to the true cords, are more hyperechoic in appearance and remain relatively immobile during phonation.

**Fig. 4:**
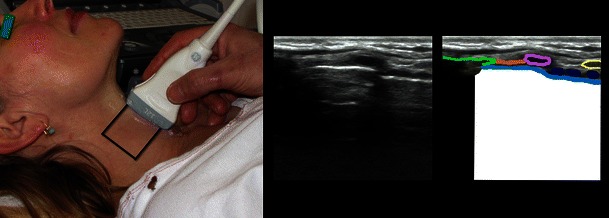
Cricothyroid membrane. *Left*: The linear high frequency transducer placed in the midsagittal plane, the scanning area is marked with a *black line. Right*: The cricothyroid membrane (*orange*); the thyroid cartilage (*green*); the cricoid cartilage (*purple*); anterior part of tracheal rings (*dark blue*); the tissue/air border (*light blue*); the isthmus of the thyroid gland (*yellow*). Below the tissue/air border only artefacts are seen (*white*)

**Fig. 5:**
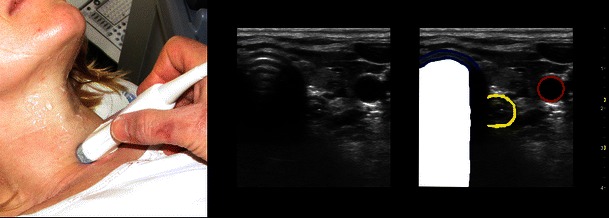
Oesophagus and trachea. Transverse scan just cranial to the suprasternal notch and to the patient’s left side of the trachea. Anterior part of tracheal cartilage (*dark blue*); oesophagus (*yellow*); carotid artery (*red*). Below the tissue/air border in the trachea only artefacts are seen (*white*)

*The cricothyroid membrane* and *cricoid cartilage* (Fig. 4) [21], *trachea* [19] [21], *oesophagus* (Fig. 5) [19, 28], *lower trachea and bronchi*: trans-oesophageal US can display a part of the lower trachea and large vessels anterior to it, that were normally a ‘blind spot’ in traditional ultrasonography. When a saline-filled endotracheal balloon is introduced during cardiopulmonary bypass, it is possible to perform ultrasonography through the trachea, with good visualisation of the proximal aortic arch and the innominate artery [29]. The bronchial wall and its layers can also be visualised from within the airway by passing a flexible ultrasound-probe through the working channel of a flexible bronchoscope or by the use of dedicated endobronchial ultrasound bronhoscopes with built-in ultrasound transducers. This technique, endobronchial ultrasound (EBUS), allows a reliable differentiation between airway infiltration and compression by tumour [30].

The *stomach* can also be visualised for evaluation of prandial status [31, 32].

#### An approach to the lower airways: pleura and lungs

In order to make a complete LUS examination, one has to scan all areas of the pleura and lungs, which can be visualised, by trans-thoracic scanning. This is both time-consuming and requires good patient cooperation during the examination, factors which are not always compatible with settings such as the emergency department, intensive care unit or the operating suite. Focused LUS, for diagnosing acute conditions, can be performed quickly and requires only minimal patient cooperation. Despite the recent publication of international consensus guidelines concerning the use of point-of-care LUS [6], there is no international consensus on how to perform focused LUS. The following approach is partly based on the principles described by Lichtenstein et al. [7] and partly on the author’s own findings [8].

**Fig. 6:**
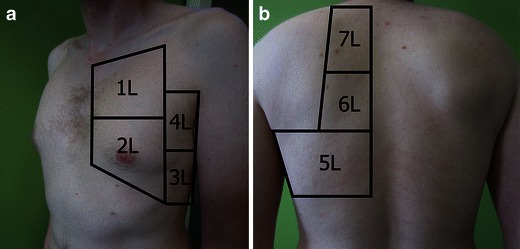
Scanning protocol used for a focused LUS examination. The anterior and posterior axillary line can be used to divide each hemithorax into an anterior, lateral and posterior surface. **a** The anterior and lateral surfaces on each side of the chest are both divided into an upper and lower quadrant, the image demonstrates the four quadrants on the left hemithorax (*1L–4L*). **b** The posterior surfaces on each side were divided into an upper, middle and lower quadrant, the image demonstrates the four quadrants on the left hemithorax (*5L–7L*). Each quadrant represents a scanning zone, in which the transducer is placed approximately in the middle of the zone making a cross sectional image of an intercostal space and the underlying pleura blades

Each hemithorax can be divided into an anterior, lateral and posterior surface, which can be further subdivided into smaller squares, representing a scanning zone (Fig. 6), which should be assessed using US. With the patient in the supine position, the transducer is quickly placed in a longitudinal axis over an intercostal space in each of the scanning zones. In each scanning zone it is noted whether pneumothorax, interstitial syndrome, lung consolidation or pleural effusion is present, or whether only normal findings are present. Once the anterior and lateral surfaces have been scanned, the patient is asked to sit up and the scanning zones on the posterior surface are assessed using the same principles. If the patient is not able to sit up, the posterior surfaces can either be scanned with the patient lying on the side or, alternatively, the transducer can be inserted in between the mattress and the patient, making it possible to scan at least a part of the posterior surface. Scanning in the B-mode is sufficient for this focused approach, but other modalities such as M-mode or colour Doppler can be employed in cases where there is doubt whether lung sliding is present or not (*see* section on pneumothorax).

#### Normal ultrasonography findings in the lower airway: pleura and lung

**Fig. 7:**
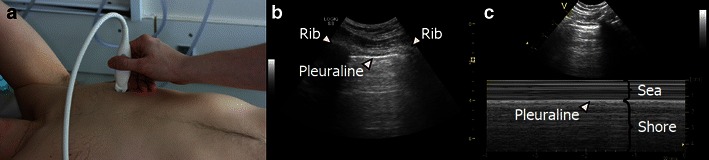
Normal LUS findings: **a** The transducer is placed in a longitudinal axis over an intercostal space at the anterior surface of the chest. **b** In the corresponding B-mode image, two ribs are visible aligning the intercostal space, as two hyperechoic lines with an underlying shadow. Lying deeper between the two ribs, a hyperechoic horizontal line is seen representing the visceral and parietal pleura. Using B-mode the movement of the *pleural line* is called “lung sliding”. **c** If M-mode scanning is applied, a characteristic pattern called “seashore sign” is visible. The *pleural line* appears as a hyperechoic line, with the more superficially placed structures appearing as *horizontal lines* similar to the sea and the part of the picture below the pleural line is grittier looking, mimicking the sand on a seashore

If the transducer is placed in a longitudinal axis over an intercostal space, superficially skin, muscle and connective tissue will be visible. The two ribs aligning the intercostal space are visible as two hyperechoic lines with an underlying shadow. Lying a bit deeper between the two ribs, a hyperechoic horizontal line is seen representing the visceral and parietal pleura (Fig. 7). Using B-mode, a horizontal motion of the pleural line can be visualised (*see* video at http://www.airwaymanagement.dk/ultrasonography-in-airway-management). The motion is synchronous with the patient’s breathing and represents the movement of the visceral pleura during inspiration and expiration. This motion is called “lung sliding” [6, 33]. If M-mode scanning is applied to a B-mode scan showing a normal pleural line with lung sliding, the corresponding characteristic M-mode image is called the “seashore sign” [7]. In M-mode, the pleural line appears as a hyperechoic line, with the more superficially placed structures (skin, muscle and connective tissue) appearing as horizontal lines similar to the sea and the part of the picture below the pleural line is more gritty looking, thus mimicking the sand on a seashore [7].

#### The diaphragm

The diaphragm and its motion can be imaged by placing a convex transducer in the subxiphoid window [34] at the mid-upper abdomen, just beneath the xiphoid process and the lower margin of liver. The transducer is tilted 45° cephalically and bilateral diaphragm motion is noticed [34]. The bilateral diaphragm will move towards the abdomen when the lungs are ventilated and towards the chest during the relaxation phase. The liver and spleen movements represent the whole movement of the right and left diaphragm during respiration, and can be visualised by placing the probe in the longitudinal plane along the right anterior axillary line and along the left posterior axillary line respectively. The movement of the most caudal margin of the liver and spleen with respiration is measured [35].

#### The stomach

**Fig. 8:**
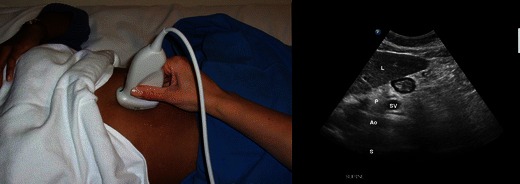
Stomach imaging. *Left*: Probe position—this shows a sagittal probe orientation in the epigastric area. *Right*: The antrum (Ant) located immediately posterior to the left lobe of the liver (*L*). The pancreas (*P*) is typically hyperechoic and located posterior to the antrum. In this figure a cross-section of the splenic vein (*SV*) may be seen as it crosses the pancreas from right to left. Posterior to the pancreas you can see a longitudinal view of the aorta (*Ao*). Spine (*S*). (Courtesy of Anahi Perlas, University of Toronto and Toronto Western Hospital, Toronto, Canada)

The stomach and its contents can be visualised by orientating the probe sagittally in the epigastric area (Fig. 8).

These techniques constitute the background for the clinical applications that are outlined in the following sections.

### Clinical use

#### Prediction of difficult laryngoscopy in surgical patients

**Fig. 9:**
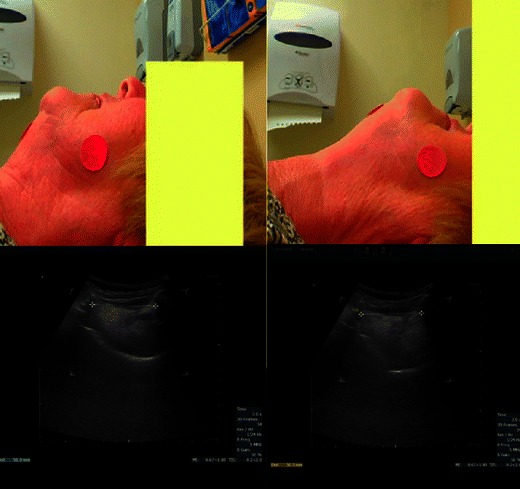
Hyomental distance ratio. The hyomental distance measured with the head in neutral position (*left*) and in maximum extension (*right*). In this case the ratio was only 1.01 and the patient correspondingly had a difficult intubation (Cormack-Lehane grade 4). (Courtesy of Jacek A. Wojtczak, Department of Anesthesiology, University of Rochester School of Medicine and Dentistry, Rochester, NY, USA)

In 50 morbidly obese patients, ultrasonographic assessment of the distance from the skin to the anterior aspect of the trachea, measured at the level of the vocal cords and the suprasternal notch, was found to be significantly greater in patients with difficult laryngoscopy despite optimisation by laryngeal manipulation [36]. These findings, however, could not be reproduced when the endpoint was laryngoscopy grade *without* the use of external laryngeal manipulation for optimisation of the laryngoscopic view [37]. The hyomental distance is the distance between the upper border of the hyoid bone and the lower border of the mentum. The hyomental distance ratio (defined as this distance measured with the head in the neutral position to the head hyperextended), ultrasonographically measured, can be used to distinguish patients with easy versus difficult laryngoscopy (Cormack-Lehane grade 3 or 4). In a pilot series involving obese and morbidly obese patients, all patients with a hyomental distance greater than 1.1 had easy laryngoscopy, and those below 1.1 had difficult laryngoscopy (Fig. 9) [38]. In a small series of elective patients, combined ultrasound assessment of the base of tongue and thickness of the soft tissue of the anterior neck had a better correlation with difficult laryngoscopy than clinical tests [39]. There are, thus, several promising ultrasonographic screening methods available, but in the authors’ opinion, evidence is still too sparse to recommend these as standard screening techniques in the general surgical population.

#### Evaluation of pathology that may influence the choice of airway management technique

**Fig. 10:**
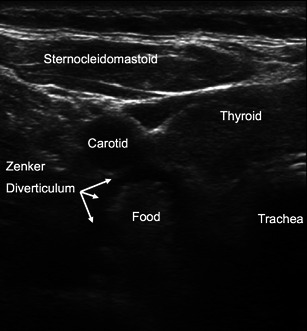
Zenker diverticulum. Transverse scan on the anterior neck above the suprasternal notch shoving a Zenker diverticulum lateral to the trachea. A bolus of solid food is seen in the diverticulum. *Sternocleid* sternocleidomastoid muscle; *Carotid* common carotid artery. (Courtesy of Peter Cheng, Kaiser Permanente Riverside Medical Center, Riverside, CA, USA)

Subglottic haemangiomas [40], laryngeal stenosis [41], laryngeal cysts [22] and respiratory papillomatosis [42] have all been sonographically described. Clinicians can be alerted to a potential source of regurgitation and aspiration when they see a pharyngeal pouch (Zenker’s diverticulum) (Fig. 10) at the posterolateral aspect of the left thyroid lobe while scanning the neck with a transverse linear high frequency probe [43]. Malignancies and their relationship with the airway can also be seen and quantified. Prenatal ultrasonography plays a crucial role in identifying fetal airway abnormalities caused by lymphatic malformations or cervical teratomas [44] and expectant management of fetal tracheolaryngeal airway obstruction can be planned.

Submandibular and parotid gland swelling has been reported after laryngeal mask insertion, especially with increases in intracuff pressures. Asymptomatic sialolithiasis occurs in between 1 per 10,000–20,000 people and the sonographic finding of salivary stones as high level reverberation echoes accompanied by posterior acoustic shadows may forewarn the clinician to avoid laryngeal mask airway insertions in patients with salivary colic and sialolithiasis [45].

#### Assessment of obstructive sleep apnea

Obstructive sleep apnea (OSA) is often a contributor to difficulties in managing the upper airway and ultrasound measurement of the width of the tongue base has been found to correlate with the severity of sleep-related breathing disorders, including the patient’s sensation of choking during the night [15]. Patients with obstructive sleep apnea (OSA) have significantly thicker lateral pharyngeal walls on ultrasonic measurement compared to patients without OSA [18].

#### Evaluating prandial status

**Fig. 11:**
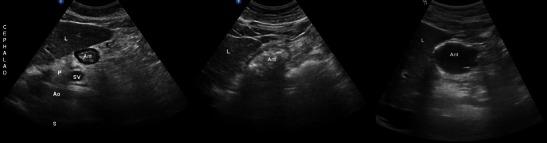
The stomach. *Left* Empty state (low aspiration risk). The empty antrum (*Ant*) appears as a small round or oval structure. It may resemble a “bull’s eye target”. When the antrum is empty, all you can see is gastric wall. What appears to be a small amount of content is actually the thickness of all the layers of the gastric wall. The gastric wall has five distinct sonographic layers. The most prominent layer can be clearly seen in this figure as a hypoechoic “ring” and it corresponds histologically to the muscularis propriae of the stomach. The antrum is located immediately posterior to the left lobe of the liver (*L*); the pancreas (*P*); splenic vein (*SV*); aorta (*Ao*); spine (*S*). *Middle*: Solid content (high aspiration risk) Solid content in the stomach appears as non-homogeneous, mostly hyperechoic content. There is usually some amount of air mixed with the solid meal, and this produces multiple “ring down artefacts” that obscure the posterior wall. We call this type of image a “frosted glass pattern”, and we can see some degree of this type of artefact in this image. *Right* Clear fluid content. Clear fluid in the stomach (such as water, tea or normal gastric secretions) can be seen as a homogeneous hypoechoic content within the antrum. When clear fluid is seen, it may be useful to do a volume estimation to better assess aspiration risk. (Courtesy of Anahi Perlas, University of Toronto and Toronto Western Hospital, Toronto, Canada)

Both experimental and clinical data show that US can detect and quantify gastric content [32] (Fig. 11). In fasting versus non-fasting subjects, US was specifically reliable in identifying a full stomach but only moderately reliable in identifying an empty stomach [46]. The cross-sectional area of the gastric antrum of healthy volunteers were found to correlate with ingested volumes of up to 300 ml fluid, especially when placed in the right lateral decubitus position[31]. Gastric outlet obstruction can also be visualised trans-abdominally on US in some patients [47]. Immediately prior to urgent endotracheal intubation in ICU patients, ultrasound scanning enabled useful identification and quantification of stomach fluid in patients. This was achieved by a mid-torso left mid-axillary longitudinal scan that identified the spleen and left hemidiaphragm; the transducer was then angled anteriorly to obtain multiple tomographic planes of the stomach’s left upper quadrant, supplemented with sagittal plane scanning [48]. It is possible to determine the nature of gastric fluid (nil, clear fluid, thick/solid) by following a standardised scanning protocol [49].

#### Prediction of the appropriate diameter of endotracheal, endobronchial or tracheostomy tube

In children [50] and young adults, ultrasound can reliably measure the diameter of the subglottic upper airway [51], demonstrating good correlation with the “gold standard” of MRI.

US can also be utilised to measure the diameter of the left main-stem bronchus, thus guiding size-selection of a left-sided double lumen tube immediately before anaesthesia. The ultrasonic measurements of the outer diameter of the trachea were performed transversely just above the sternoclavicular joint. The ratio between the diameter of the trachea and of the left main-stem bronchus was obtained by examining the CT images of a series of patients. The ratio between left main-stem bronchus diameter on CT imaging and outer tracheal diameter measured with US was 0.68. These results were found to be comparable with those obtained using chest radiograph as a guide for selecting left double lumen tube size [52, 53]. In tracheostomised children, the size and shape of a potential replacement tracheostomy tube [54] can be determined by ultrasonographically measuring the tracheal width and the distance from the skin to the trachea. Adequate images can be obtained by placing the ultrasound probe just superior to the stoma.

#### Localisation of the trachea

**Fig. 12:**
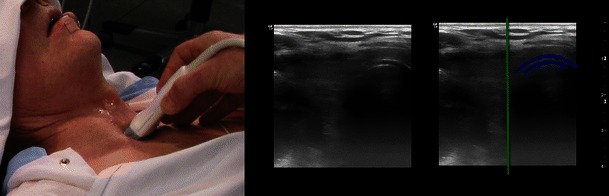
Localisation of the displaced trachea. The patient previously had surgery and radiation therapy for neck cancer and no structures could be palpated. *Left*: The transducer is placed transversely in the midline over the suprasternal notch. *Middle*: The scanning image. *Right*: The cartilage of the tracheal ring (*dark blue*) is deviated to the patient’s left side. The *green line* indicates the midline of the neck

Accurate localisation of the trachea is challenging in the presence of obesity, a short thick neck, prior irradiation or neck surgery, neck masses and any thoracic pathology causing tracheal deviation. Chest radiographs and needle aspiration techniques to locate the trachea may be futile in such circumstances [55]. Preoperative US for localisation of the trachea (Fig. 12) is especially useful in emergency cases and where awake tracheostomy is chosen because of a predicted difficult mask-ventilation[55] or difficult tracheal intubation.

#### Localisation of the cricothyroid membrane

The cricothyroid membrane plays a crucial role in airway management but conventional identification by anaesthetists based on surface landmarks and palpation only correctly identifies it in 30 % of cases [56]. Ultrasonography allows reliable [56] and rapid [57] identification of the cricothyroid membrane and trachea prior to both elective trans-tracheal cannulation and emergency cricothyrotomy as demonstrated by an obese patient with Ludwig’s angina, in whom it was not possible to identify the trachea by palpation, whose trachea was eventually located 2 cm lateral to the midline using a portable ultrasound machine [58]. In this way the localisation of the trachea allows the clinician to approach the difficult airway either by placing a trans-tracheal catheter or performing a tracheostomy prior to anaesthesia, or by performing an awake intubation (but with the added safety of having localised the cricothyroid membrane in advance in case the awake intubation fails and emergent transcricoid access becomes necessary). One method for localising the cricothyroid membrane is described as follows using a 10-MHz linear array probe: A transverse, midline scan is performed from the clavicles to the mandible. The cricothyroid membrane is identified by its characteristic echogenic artefact, with the cricothyroid muscles lateral to it, and the thyroid cartilage cephalad. In a study on 50 emergency department patients, the cranio-caudal level of the cricothyroid membrane was located by a longitudinal sagittal midline scan followed by sliding the probe bilaterally to localise the lateral borders of the cricothyroid membrane. The mean time to visualisation of the cricothyroid membrane was 24.3 s [57].

**Fig. 13:**
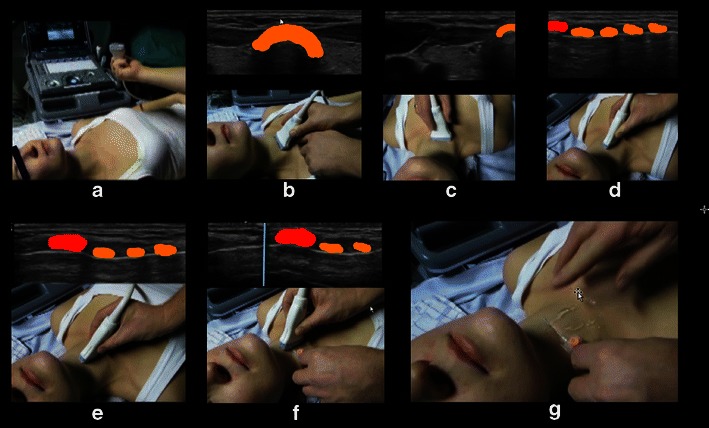
Localisation of the cricothyroid membrane. *Orange* is the anterior part of the tracheal rings. *Red* is the anterior part of the cricoid cartilage. The *turquoise line* is the shadow from the needle that has been slid underneath the transducer and placed just cranial to the cricoid cartilage, thus indicating the position of the lower part of the cricothyroid membrane, where an emergency airway access should be performed. **a** The patient is lying supine and operator stands on the patient’s right side facing the patient. **b** The linear transducer is placed transversely over the neck just above the suprasternal notch and the trachea is observed in the midline. **c** The transducer is moved to the patient’s right side so that the right border of the transducer is superficial to the midline of the trachea. **d** The right end of the transducer is kept in the midline of the trachea while the left end of the transducer is rotated into the sagittal plane, resulting in a longitudinal scan of the midline of the trachea; the caudal part of the cricoid cartilage is seen (*red*). **e** The transducer is moved cranially and the cricoid cartilage (*red*) is seen as a slightly elongated structure that is significantly larger and more anteriorly than the tracheal rings. **f** A needle is moved under the transducer from the cranial end, used only as a marker. The shadow (*turquoise line*) is just cranial to the cranial border of the cricoid cartilage. **g** The transducer is removed and the needle indicates the distal part of the cricothyroid membrane

A simple and systematic approach to localising the cricothyroid membrane is shown in Fig. 13.

#### Airway-related nerve blocks

Ultrasonography can be applied to identify and potentially block the superior laryngeal nerve as part of the preparation for awake fibre-optic intubation when the landmarks for palpation are difficult to identify. The greater horn of the hyoid bone and the superior laryngeal artery were identified and the local analgesic was injected in between in this case report of a patient with cervical spine disease [59]. The nerve itself can be elusive and not easily identified sonographically. In 100 ultrasound examinations for the superior laryngeal nerve space (the space delineated by the hyoid bone, the thyroid cartilage, the pre-epiglottic space, the thyrohyoid muscle and the membrane between the hyoid bone and the thyroid cartilage), all components of the space were seen in 81 % of cases, whereas there was a suboptimal, but still useful, depiction of the space in the remaining 19 % of cases. The superior laryngeal nerve itself was not seen in that series [60]. However, recent reports suggest ultrasound-guided superior laryngeal nerve block in humans may become feasible. Using a hockey stick-shaped 8- to 15-MHz transducer, ultrasonographic visualisation of the superior laryngeal nerve was described in 20 volunteers and successfully confirmed by US-guided in-plane injection of green dye in two cadavers [61].

#### Confirmation of endotracheal tube placement

The tracheal tube entering the trachea or the oesophagus can be confirmed by (1) direct performance of real time scans of the anterior neck during intubation, (2) indirectly looking for ventilation at the pleura or diaphragmatic level, or (3) by combining these techniques. Accidental oesophageal intubation can be recognised immediately when US scans are done real-time, and corrected before ventilation is initiated and before air is forced into the stomach, resulting in an increased risk of emesis and aspiration. Looking for ventilation at the pleural level has the advantage of distinguishing between tracheal and endobronchial intubation at least to some extent. Both the direct and the indirect confirmation are advantageous over capnography as they can be applied in the very-low cardiac output situation. Ultrasonography is also better than auscultation in noisy environments, such as helicopter retrievals. In a cadaver model where a 7.5-MHz curved probe was placed longitudinally over the cricothyroid membrane, it was possible for residents given only 5 min of training in the technique to correctly identify oesophageal intubation (97 % sensitivity) when this was performed *during* the intubation, dynamically. When the examination was performed *after* the intubation, the sensitivity was very poor [62].

Accidental oesophageal intubations were detected in all five incidents among 40 elective patients when a 3–to 5-MHz curved transducer was placed at the level of the cricothyroid membrane aimed cranially at 45°. Tracheal passage of the tube was seen as a brief flutter deep to the thyroid cartilage, whereas oesophageal intubation created a clearly visible bright (hyperechoic) curved line with a distal dark area (shadowing) appearing on one side of, and deep to, the trachea [63].

By placing a linear probe transversely on the neck just superior to the suprasternal notch, it was possible to differentiate tracheal from oesophageal intubations in 33 patients with normal airways, and in another 150 patients intubated either in the trachea or in the oesophagus in random order [64]. In a controlled operating room setting, skilled ultrasonographers have been able to consistently detect passage of a tracheal tube into either the trachea or oesophagus in normal airways [65]. Using a portable hand-held ultrasound machine, indirect confirmation of tracheal tube placement in 15 patients was possible by scanning the third and fourth intercostal spaces bilaterally during pre-oxygenation, apnoea, bag-mask ventilation, intubation and positive pressure ventilation post-intubation [66]. Furthermore, colour Doppler was used as a supplement to observe lung sliding, confirming lung ventilation [66].

The distinction between tracheal and endobronchial intubation can be made by scanning the lung bilaterally. If there is lung sliding on one side and lung pulse (*see* “Pneumothorax” section) on the other side, it indicates that the tip of the tube is in the main-stem bronchus on the side where lung sliding is observed. The tube should be withdrawn until lung sliding is observed bilaterally, indicating that the tip of the tube is again placed in the trachea [67]. Indirect confirmation of intubation by detection of lung sliding was studied in fresh cadavers where the tip of the tube was placed either in the oesophagus, trachea or right main-stem bronchus. A high sensitivity (95–100 %) was found for detection of oesophageal versus airway (trachea or right main-stem bronchus) intubation. There was lower sensitivity (69–78 %) in distinguishing right main-stem bronchus intubation from tracheal intubation, possibly due to transmitted movement of the left lung from right lung expansion [68]. Plain film radiographs are considered the reference method for detecting endobronchial versus endotracheal intubation in the intensive care unit [69] and are therefore assumed to have a sensitivity and a specificity of 100 %. In the anaesthesia or emergency setting, it often takes too long to get a plain film radiograph and there is a need for alternative methods that can be applied without delay.

In paediatrics, indirect confirmation of tracheal versus oesophageal intubation can be distinguished by depicting diaphragmatic movement bilaterally [34]. However, when the technique is used to distinguish between main-stem bronchus versus tracheal intubation, chest radiography is better than diaphragmatic ultrasound [70]. The *combination* of direct transverse neck scan at the cricothyroid membrane and detection of lung sliding on lung ultrasound correctly detected three cases of oesophageal intubation despite four cases of pneumohaemothoraces in 30 emergency department patients [71], and in patients with difficult laryngoscopy in the clinical emergency setting [72]. Filling the tracheal cuff with fluid helps in visualising the cuff position [73], but a metal stylet does not augment visualisation of the endotracheal tube [74]. Passing of the tracheal tube is visible in all children and characterised by the widening of the vocal cords when the transducer is placed at the level of the glottis [75]. Ultrasonography is also useful in confirming the correct position of a double-lumen tube [52, 76].

##### Authors recommendations

By performing a transverse scan above the sternal notch over the trachea, the location and appearance of the oesophagus can be noted. Intubation should then be performed. If the tube is seen passing into the oesophagus, remove it without ventilating the patient and make another intubation attempt, possibly using another technique. If the tube is not seen, or if it is seen in the trachea: ventilate the patient via the tube. Move the transducer to the midaxillary line, and look for lung sliding bilaterally. If there is bilateral lung sliding, it is confirmation that the tube is in the airway, but intubation of a main-stem bronchus cannot be ruled out. If there is one-sided lung sliding and lung pulse on the other side, then main-stem intubation is likely, and the tube should be withdrawn gradually until bilateral lung sliding is present. If there is no lung sliding on either side, but a lung pulse, there is a small risk of the tube having entered the oesophagus. If there is neither lung pulse nor lung sliding, then a pneumothorax should be expected. In experienced practitioners verification of endotracheal tube placement is as fast as auscultation alone and faster than the standard method of combined auscultation and capnography [77, 78].

#### Confirmation of laryngeal mask placement

Perioperative ultrasonography is able to replace fibre-optic examination for confirmation of the correct placement of a laryngeal mask airway (LMA) in assessing adequacy of the laryngeal seal and pulmonary ventilation. In 31 patients undergoing general anaesthesia with an AuraOnce or AuraFlex disposable LMA, the position of the LMA cuff was confirmed by transverse neck ultrasound and reconfirmed with intra-LMA fibre-optic laryngoscopy . The ultrasound grade of LMA position was found to correlate strongly with the fibre-optic grade of LMA position (*r* = 0.92). Additionally, non-invasive ultrasound examination can further give insight into the cause of airway/ventilation events that may be interfering with the LMA placement and ventilation [79].

#### Tracheostomy

When faced with impalpable surface landmarks, accurate localisation of the trachea can be very challenging. Preoperative ultrasonography for localisation of the trachea is valuable for both surgical [55] and percutaneous dilatational tracheostomy. In children, preoperative US can determine the precise tracheostomy position, thereby preventing subglottic damage of the cricoid cartilage and the first tracheal ring, mitigating haemorrhage due to abnormally placed or abnormally large blood vessels and reducing the risk of pneumothorax [80].

#### Percutaneous dilatational tracheostomy (PDT)

Ultrasonography allows real-time [81, 82] localisation of the trachea, visualisation of the anterior tracheal wall and pretracheal tissue including blood vessels [83], and selection of the optimal intercartilaginous space for placement of the tracheostomy tube [84]. US-guided PDT results in a significantly lower rate of cranial misplacement of the tracheostomy tube than “blind” placement [84]. The distance from the skin surface to the tracheal lumen can be measured in order to predetermine the length of the puncture cannula that is needed to reach the tracheal lumen without perforating the posterior wall [85]. The distance can also be used to determine the optimal length of the tracheostomy cannula [86]. Successful US-guided PDT has been utilised when bronchoscope-guided techniques were abandoned [85]. Bronchoscope guided PDT also often results in considerable hypercapnia, whereas ultrasound Doppler-guided PDT does not [87]. Autopsy reports of cases of fatal bleeding following PDT have revealed erosion of the innominate vein and the arch of the aorta, where the tracheostomy level turned out to be much more caudal then intended. It is likely that pre-procedure ultrasonographic determination of the optimal PDT level could diminish this risk by avoiding major blood vessels [88].

In a prospective series of 72 PDTs, the combination of US and bronchoscopy was applied. All subjects had their pretracheal space examined with US pre-procedurally, leading to a change in the planned puncture site in 24 % of cases and to a change of the procedure to a surgical tracheostomy in one case where a goitre with extensive subcutaneous vessels was discovered on ultrasound examination [89]. In a different approach, when a small curved transducer was used in the transverse plane to localise the trachea in the midline, then turned longitudinally to allow in-plane needle puncture, this enabled the needle course to be followed from the skin surface to the trachea. After guide-wire insertion, CT scans that were performed showed that although all punctures successfully entered the trachea in first (89 %) or second (11 %) attempt, the guide-wire was placed laterally to the ideal midline position in five of nine cadavers [90]. Another approach using real-time ultrasonic guidance with a linear high frequency transducer placed transversely over the trachea was more successful and resulted in visualisation of the needle path and satisfactory guide-wire placement in all 13 patients.[82]

#### Evaluation of lung pathology

The most common lung pathology visualised by LUS can roughly be divided into the following ultrasonography morphological patterns: pneumothorax, interstitial syndrome, focal B-lines, lung consolidation, atelectasis and pleural effusion.

#### Pneumothorax

**Fig. 14:**
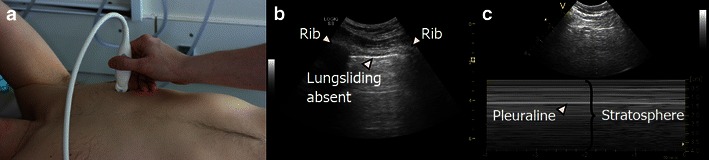
Pneumothorax. **a** The transducer is placed in a longitudinal axis over an intercostal space at the anterior surface of the chest; this is the area where free air in the chest cavity is expected to be located. **b** If pneumothorax is present the pleura-line only represents the parietal pleura, therefore the motion of the visceral pleura cannot be visualised and subsequently lung sliding is absent. **c** In M-mode the lack of lung sliding will appear as the “stratosphere sign”, a pattern only consisting of horizontal lines

Generally, LUS is a better diagnostic tool than conventional chest X-ray for the diagnosis of pneumothorax, especially for ruling out pneumothorax [6]. In meta-analysis, LUS examinations have a pooled sensitivity of 78.6–90.9 % and specificity of 98.2–98.4 % in diagnosing pneumothoraces in supine patients, compared with chest radiographs (39.8–50.2 % and 99.3–99.4 % respectively) [91, 92]. Four distinct characteristic ultrasound signs have been described for the diagnosis and exclusion of pneumothorax: lung sliding, B-lines, lung pulse and lung point [6, 93]. When diagnosing pneumothorax with LUS (Fig. 14), the characteristic signs can pragmatically be divided into these three categories:

- Findings which exclude pneumothorax
- Findings which are suggestive of pneumothorax
- Findings which are diagnostic of pneumothorax

**Fig. 15:**
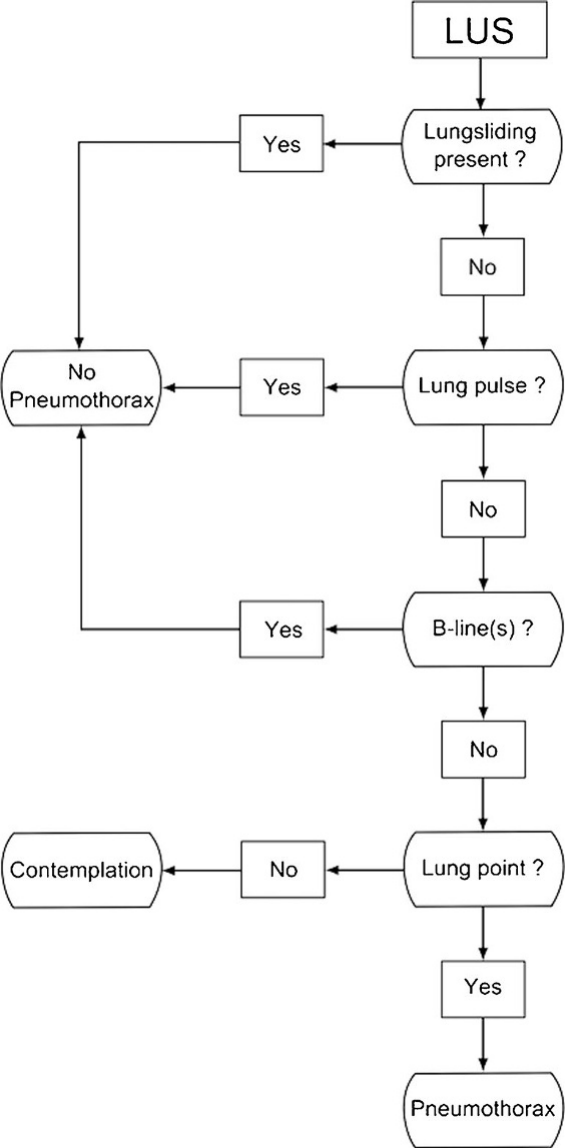
Algorithm for the diagnosis and exclusion of pneumothorax using LUS. Initially one should look for signs which rule out the presence of pneumothorax [lung sliding, lung pulse, B-line(s)] at the anterior surface of the chest. If none of these are present, then one should gradually move the transducer laterally and posterior on the surface of the chest and look for lung point in order to establish the diagnosis of pneumothorax. If neither signs are present, contemplation is needed since pneumothorax can neither be ruled in nor out. In young, previously healthy patients, such as most trauma patients, the absence of lung sliding alone is sufficient to diagnose pneumothorax. In such patients the absence of all signs will be consistent with pneumothorax. In comparison, patients with known lung diseases or previous chest surgery may have a variety of causes for the absence of lung sliding. In such patients the absence of all signs can neither be used to rule in nor to rule out a pneumothorax and further imaging should be performed in order to establish whether pneumothorax is present or absent

The three categories are discussed in detail below, an algorithm for the diagnosis of pneumothorax using LUS is given in Fig. 15. The algorithm can be used in most clinical settings and scenarios, even though in a trauma setting a more simple approach involving the presence or absence of lung sliding may be used instead.

##### Findings which exclude pneumothorax

Lung sliding and B-lines can only be visualised when the two pleural blades are in contact with each other. In the supine patient, any free air in the pleural cavity will tend to rise and accumulate just below the anterior chest wall. Hence, when scanning the anterior surface of a supine patient’s chest, the presence of lung sliding or B-lines excludes a pneumothorax on the side that is examined [33, 94–101].

In intubated patients where the tube accidentally has been placed in the main-stem bronchus, one will often be able to visualise a phenomenon called “lung pulse” in the non-ventilated lung [67, 102]. Lung pulse is seen as a discrete movement of the pleural line, in synchrony with the heart beat. This occurs due to the transmission of heart movements to the lung and the visceral pleura. Lung pulse can only be seen when the two pleural blades are in contact which each other, therefore the presence of lung pulse also rules-out a pneumothorax [102].

##### Findings which are suggestive of pneumothorax

When a pneumothorax exists, the visceral and parietal pleura will not be in contact with each other, and lung sliding will be absent [33, 94–101]. The absence of lung sliding is not pathognomonic of a pneumothorax as lung sliding can also be absent when pleural adhesions, lung bullae, apnea, or inadvertent intubation of the opposite main-stem bronchus [95, 102, 103]. In trauma patients, these concomitant conditions may be exceedingly rare. Several studies have affirmed that the absence of lung sliding on the anterior chest surface of a non-intubated, spontaneously breathing trauma patient can be used as a diagnostic sign of pneumothorax [96–101]. However, in intubated patients or in whom underlying lung diseases may exist, the absence of lung sliding is not diagnostic, but should prompt the clinician to still consider the diagnosis of pneumothorax [95].

##### Findings which are diagnostic of pneumothorax

When patients are in the supine position, any air in the pleural cavity will tend rise to just below the anterior surface of the chest. Depending on the size of the pneumothorax, there might be no air between the two pleural blades more laterally or posteriorly; and in these areas, lung sliding will be present. The boundary between an area where the pleura blades are in contact and an area with air in between the blades will move in conjunction with the patients breathing. With LUS, the change from no-air to air between the pleural blades can be directly visualised. If the transducer is placed in such a transition zone during the patient’s respiratory cycle, the US image will change from lung sliding to no lung sliding. The visualisation of such a transition is known as “lung point”, which is considered a diagnostic, pathognomonic, sign of pneumothorax [95, 99, 104] (*see* video at http://www.airwaymanagement.dk/ultrasonography-in-airway-management). When the patient is in the supine position, lung point can be found using the following method: The transducer is placed on the patient’s anterior chest wall, in the longitudinal axis over an intercostal space. If lung sliding is present, pneumothorax can be ruled out. If lung sliding is absent, the transducer is rotated to the horizontal axis over the intercostal space. The transducer is then gradually moved along the intercostal space laterally and further posteriorly. If a change occurs (from absent lung sliding to its presence), the lung point has been identified. Not all patients with pneumothorax have a lung point; the sensitivity for detecting a pneumothorax by observing a lung point is therefore lower than by observing the absence of lung sliding [95, 99, 104].

##### Quantification of pneumothorax size

The relative location of a lung point on the chest could theoretically be used to quantify the size of a pneumothorax [93]. It is more anteriorly situated in a small pneumothorax and nearer the posterior surface of the chest if the pneumothorax is larger in size. Studies suggest that LUS can adequately quantify large and small pneumothoraces, but not moderately sized ones [96, 99]. It is notable that no consensus could be reached on whether LUS could accurately quantify pneumothorax or not, in the published consensus article on LUS [6]. Hence, despite the well-established diagnostic accuracy of LUS for the diagnosis of pneumothorax, no evidence-based guidelines have been established on how to use LUS for the quantification of pneumothorax and the role of concurrent supplementary imaging such as plain chest X-ray or CT. It is the authors’ opinion that in a critically ill patient showing clinical signs of pneumothorax, with LUS findings consistent with this diagnosis, one should perform acute chest tube insertion in order to provide acute treatment of the patient and not await supplementary imaging. In a clinically stable patient with LUS showing signs of pneumothorax, one should await other forms of diagnostic imaging in order to establish the size of the pneumothorax prior to initiating treatment. Even though the quantification of pneumothorax size by the use of LUS is controversial, studies have demonstrated that LUS is a reliable tool for monitoring whether the size of a pneumothorax is increasing or decreasing [105, 106].

#### Interstitial syndrome (IS)

The presence of IS has a very high sensitivity, but not specificity, for the diagnosis of cardiogenic pulmonary oedema. The latter is because many other conditions can also cause IS; e.g. non-cardiac pulmonary oedema, drowning, acute respiratory distress syndrome (ARDS), bilateral interstitial pneumonia and interstitial lung disease [107–121]. LUS can accurately locate IS in the lung lobes when compared with CT [122, 123].

**Fig. 16:**
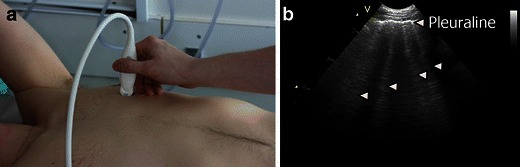
Multiple B-lines. **a** The transducer is placed in a longitudinal axis over an intercostal space. **b** B-lines (*arrowheads*) are seen as strong hyperechoic, laser-like, *vertical lines* originating from the pleura and extending to the bottom of the field of view without decreasing in intensity. B-lines move synchronously with lung sliding

By systematically scanning two anterior, two lateral and three posterior zones, IS is defined by the presence of both of the following criteria[6, 8]: (1) a positive scanning zone, defined by the presence of three or more B-lines (Fig. 16) (*see* video at: http://www.airwaymanagement.dk/ultrasonography-in-airway-management) in a longitudinal plane between two ribs; (2) at least two zones have to be positive on each side when scanning the anterior and lateral zones. Any findings on the posterior surface are not included in the definition of IS [6].

##### Cardiogenic IS

Cardiogenic pulmonary oedema is a common cause of IS in emergency department or intensive care patients admitted acutely with dyspnoea [107–112]. Typically, the location of the B-lines follows the effects of gravity and is therefore often more pronounced in the posterior and lateral scanning zones, rather than the anterior zones. The pleura line should appear normal and intact [107–112]. B-lines are highly dynamic in cardiogenic pulmonary oedema [124, 125]. Due to its high sensitivity in detecting IS, the absence of IS on LUS can be used as a pivotal diagnostic tool in ruling out cardiogenic pulmonary oedema [107–112]. Absent B-lines has also been suggested as a fast way to rule out cardiogenic shock [126]. In both cardiogenic and non-cardiogenic IS, the change in the total number of B-lines has a potential role as a monitoring tool [5, 114, 120, 127–130]. If the total number of B-lines seen when performing diagnostic LUS is counted, then the total number seems to correlate to pulmonary artery systolic pressure, pulmonary vascular resistance, extravascular lung water and prognosis [131–133].

##### Non-cardiogenic IS

IS has been described in various interstitial lung diseases, ARDS, pneumonia and non-cardiogenic pulmonary oedema [113–121]. Often, the location of the B-lines does not follow gravitational rules in many of these conditions. The pleural line may appear thickened and fragmented, some spared areas may be devoid of B-lines, and subpleural consolidations may be seen [113, 115, 117, 119, 134–136]. In other cases of pulmonary oedema from fluid overload and renal failure, the LUS findings will be similar to those of cardiogenic pulmonary oedema [114, 116, 120, 121, 134–136]. Hence, when IS is detected, supplementary focused echocardiography can be employed in order to establish whether the IS is of cardiogenic origin or not [121]. Even though LUS has a high sensitivity for the detection of cardiogenic pulmonary oedema, CT still has a higher sensitivity for the detection of many of the conditions causing non-cardiogenic IS, especially interstitial lung disease. Hence a LUS with normal findings cannot be used to rule out conditions such as interstitial lung disease, and supplementary CT should still be considered since it has a pivotal role in diagnosing, staging and monitoring of these diseases [137].

#### Focal B-lines

Occurrence of multiple, isolated, B-lines can both be a normal and pathological sign [6]. Of patients with normal chest imaging, 21–28 % have multiple B-lines in the lower lateral intercostal space [2, 109, 138]. Focal areas with multiple B-lines can also be seen in any disease with localised increased density of the lung tissue, e.g. lower lobe pneumonia [109, 138]. If the density increases and the lung tissue becomes filled with fluid, the pattern will change to that of lung consolidation (*see* section below). Other causes of focal B-lines are pneumonitis, atelectasis, pulmonary contusion, pulmonary embolism, pleural disease and malignancy [6].

#### Lung consolidation

**Fig. 17:**
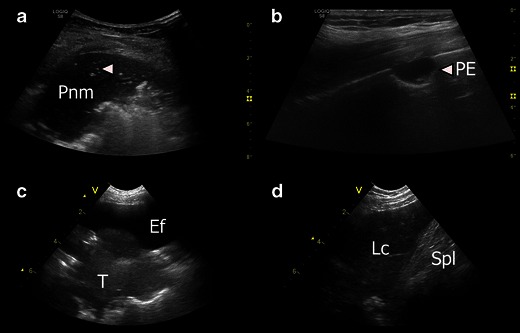
**a** Consolidation: A diffusely demarcated consolidation (*Pnm*) with an appearance similar to liver tissue. Air bronchograms (*arrowhead*) are visible within the consolidation. **b** Lung consolidation due to pulmonary embolism. A sharply defined hypoechogenic consolidation (*PE*) without air bronchograms just below the pleural line. **c** Tumour. Just below a pleural effusion (*Ef*), a relatively well demarcated tumour (*T*) can be seen located on the surface and within the lung tissue. **d** Lung contusion. US image from a patient who had suffered blunt trauma to the left side of the chest. LUS revealed multiple rib fractures and an underlying lung contusion (*Lc*) just cranially to the spleen (*Spl*)

The most common causes of lung consolidation (Fig. 17) are pneumonia and pulmonary embolism. A tumour often has a similar appearance on ultrasonography, which is why it is also mentioned in this section. LUS is able to confirm or negate the presence of pneumothorax, IS and pleural effusion. LUS can rule-in lung consolidation, but since LUS cannot visualise the entire lung surface and a lung consolidation does not necessarily “touch” the pleura, LUS cannot rule-out lung consolidation [6]. Thus, LUS can accurately diagnose pneumonia, pulmonary embolism, lung contusion and lung/pleural tumour, but a normal LUS cannot exclude the presence of any of these conditions [6]. Using the different sonomorphological patterns, LUS allows the clinician to differentiate between different causes of lung consolidation [139–141]. In a study of patients with pleuritic chest pain and a normal chest X-ray, LUS could detect “radio-occult” lung consolidation in a substantial proportion of the included patients [142]. Hence, in patients with pleuritic chest pain and a normal chest X-ray, LUS is an essential diagnostic tool [143]. Even in critically ill patients, LUS can detect lung consolidation with a 99 % feasibility and exhibit good correlation with CT [144]. Studies assessing LUS’s inter- and intra-observer agreement for the detection and differential diagnosis between the different types of lung consolidation are scarce, this area needs to be further addressed in future studies. A summary of the most common characteristic ultrasonographic appearances of the different types of lung consolidation and atelectasis are given in Table 1. The ultrasonographic appearances may vary in the mentioned conditions; the characteristics presented in Table 1 should therefore be seen as a rough guide rather than a comprehensive list.

**Table 1:** Common sonomorphological appearance of atelectasis and common causes for lung contusion

Condition	Echogenecity	Demarcation	Bronchograms	Miscellaneous
Pneumonia	Hyperechoic	Diffuse^a^	Air bronchograms present	
Pulmonary embolism	Hypoechoic	Sharp	Absent	Triangular/rounded shape
				Multiple lesions may be present
				Often minor pleural effusion present
Tumour	Hypoechoic^b^	Diffuse/sharp	Absent	Abnormal vessel supply may be present
				Visible growth into or destruction of organs and anatomical structures may be present
Lung contusion	Hyperechoic	Diffuse^a^	Air bronchograms may be present	
Compression atelectasis	Hyperechoic	Sharp	Absent	Wedge shape
				Adjoining pleural effusion present
				“Jelly fish” sign
				Re-ventilation during inspiration may be present
Obstruction atelectasis	Hyperechoic	Diffuse^a^	Fluid bronchograms present	No or minimal pleural effusion present
				No re-ventilation during inspiration present

^a^If larger areas of the lung are affected such as an entire lobe, the demarcation appears sharp, corresponding to the anatomical structures

^b^Tumours are most often hypoechoic, but may also appear hyperechoic or have areas with different echogenicity

##### Pneumonia

The characteristic ultrasonographic appearance of pneumonia is a diffusely demarcated, hypoechoic lung consolidation. Visible air bronchograms within the consolidation is a very characteristic finding of pneumonic lung consolidation, typically not seen in the other causes of lung consolidation. The air bronchograms are seen as punctate or linear hyperechoic structures within the lung consolidation [145–149]. In a multicentre study of LUS for diagnosing pneumonia, the sensitivity was 93.4 % and specificity 97.7 % [148]. In children, the diagnostic accuracy of LUS in diagnosing pneumonia makes it a potential alternative to conventional chest X-ray [150–153]. In the emergency department setting, LUS has been used to differentiate between viral and bacterial pneumonia [154, 155]. LUS can also be used as a follow-up tool in pneumonia [147, 156–158].

##### Pulmonary embolism

A meta-analysis of LUS’s diagnostic capabilities in the diagnosis of pulmonary embolism showed a pooled sensitivity of 80 % and specificity of 93 % [159]. The consolidation typically is a hypoechoic, triangular/round, well-demarcated area of lung consolidation, varying from millimetres to several centimetres in size. The average patient with pulmonary embolism has two to three visible lung consolidations and often a pleural effusion [160–170]. The following diagnostic criteria for the diagnosis of pulmonary embolism using LUS were proposed as part of a multicentre study for patients clinically suspected to have pulmonary embolism [166]:

- Confirmed pulmonary embolism: two or more characteristic triangular or rounded pleura-based lesions
- Probable pulmonary embolism: one typical lesion with a corresponding low-grade pleural effusion
- Possible pulmonary embolism: non-specific subpleural lesions <5 mm in size or a single pleural effusion alone

How LUS should or could be used in conjunction with other diagnostic modalities such as CT and ventilation-perfusion scan, remains to be established. Supplementary imaging should be performed if LUS findings are suggestive/diagnostic of pulmonary embolism. Whether supplementary imaging should be performed if LUS findings are normal or non-diagnostic depends on whether pulmonary embolism is clinically suspected. Even though LUS’s exact role in the diagnosis of pulmonary embolism remains to be established, it probably has a role alongside initial diagnostic tests in an emergency department. When focused LUS is used systematically in patients with respiratory symptoms admitted to an emergency department, apart from diagnosing other conditions, LUS is also able to identify patients with pulmonary embolism, which would otherwise have been missed [171].

Assessment of the right ventricle using echocardiography serves an important role in patients with pulmonary embolism. In the acute setting echocardiography is used as a part of the assessment of the need for initiating thrombolysis in the most critically ill patients [172]. In the follow-up of the patient echocardiography is used when assessing long-term complications such as the development of pulmonary hypertension [172].

Even though echocardiography serves as an important tool for assessment of treatment and prognosis, its diagnostic accuracy for diagnosing pulmonary embolism is limited, since approximately half of the patients with pulmonary embolism have normal echocardiographic findings [173]. In the same manner, ultrasonography of the deep veins in the legs also has limited diagnostic accuracy in these patients [174]. In recent published studies, the integrated use of ultrasonographic assessment of the heart, lungs and deep veins was assessed in a population of patients suspected of having pulmonary embolism and in a more unselected population of patients with respiratory symptoms admitted to an emergency department. This combination of ultrasonographic assessment of three sites for signs of pulmonary embolism yielded a high sensitivity (90.0–100 %) and specificity (86.2–88.9 %) for the detection of pulmonary embolism [171, 175]. An approach in which the site of origin (deep veins), the path (venous circulation/heart) and the end target (lungs) are all assessed using ultrasonography may prove useful.

##### Lung contusion

When lung contusion is visualised using LUS it may either be seen as areas with focal interstitial syndrome or areas with lung consolidation [176, 177]. Air bronchograms may be visible in the consolidated lung tissue [177]. In two small studies, where increased focal B-lines or lung consolidation were sonographic indicators of the presence of lung contusion, LUS had a sensitivity of 86–94.1 % and specificity of 96.1–97 % for diagnosing lung contusion in trauma patients when compared with CT. In both studies, LUS’s diagnostic capabilities was better than conventional chest X-ray [176, 177]. In theory, LUS should be able to estimate the extent of lung contusion using an approach similar to what has been described for monitoring pneumonia and recruitment manoeuvres in an intensive care setting [157, 178]. To the best of our knowledge, no studies have addressed this question; hence it remains to be investigated whether LUS has a potential role for decision making in patients with lung contusion.

##### Tumour

Tumour appearance on ultrasonography varies considerably. Most often they are seen as a hypoechoic structure, but isoechoic and hyperechoic tumours have also been described. Tumours can also have an inhomogeneous appearance with mixture of hypo-, iso- and hyper-echoic areas. The tumours may both be relatively well demarcated or diffusely demarcated. Necrosis of the tumour may be visualised using LUS. Signs of invasive growth into adjoining structures can help to establish the diagnosis of a malignant tumour [179, 180]. LUS seems to be better than CT to diagnose invasion of peripheral tumours [181, 182]. US/CT-guided biopsy is often performed to confirm the diagnosis and to establish the type of malignancy [179, 183]. US can be used as an integrated part for staging of lung cancer [183].

##### Uncharacteristic lung consolidation

Some lung consolidations do not fit into one of the patterns described above. More specialised forms of lung ultrasound with the use of Doppler, power Doppler, spectral analysis and contrast-enhanced ultrasonography (CEUS) may be of help in order to establish the type of lung consolidation [184–193].

#### Atelectasis

In atelectasis, the lung tissue is completely airless. The characteristic finding on ultrasonography of atelectasis is a homogenous, well-demarcated hyperechoic lung consolidation. In comparison with pneumonia, no air bronchograms are visible. The size of the lung consolidation may vary during the breathing cycle due to ventilation [179]. Compression atelectasis is often present together with pleural effusion. The atelectatic lung tissue is often seen “floating” in the pleural effusion, which gives a characteristic movement of the atelectasis due to ventilation and heartbeat. The moving lung atelectasis has also been called “jelly fish sign” or been compared to a waving hand. In obstructive atelectasis, the lung is filled with fluid instead of air [179]. The sonographic pattern is the same as a pneumonic consolidation, with the important difference that fluid bronochograms are visible instead of air bronchograms. The fluid bronchograms are seen as hypoechoic round areas or tube-like structures mimicking vessels. US Doppler can be used to differentiate between fluid bronchograms and blood vessels [179].

#### Pleural effusion

**Fig. 18:**
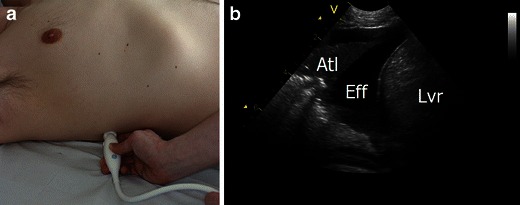
Pleural effusion. **a** The transducer is placed in a longitudinal axis over an intercostal space on the most dependent area of the chest, since this is the area where free fluid in the chest cavity is expected to be located. **b** Pleural effusion (*Eff*) often is visualised as a dark, anechoic area lying in between the visceral and parietal pleura on ultrasonography and just above the liver (*Lvr*) or spleen. Underlying compression atelectasis (*Atl*) of the lung is also often present

Pleural effusion (Fig. 18) will most often be visualised as a dark, anechoic area lying in between the visceral and parietal pleura on ultrasonography [194–199]. LUS cannot accurately estimate whether an effusion is a transudate or an exudate. Neither can LUS rule out a parapneumonic effusion or empyema. Hence in a pleural effusion of unknown origin, LUS cannot replace a diagnostic thoracocentesis [6, 195, 199]. However, some characteristic US findings can be used as a guide until the results of the diagnostic thoracocentesis become available. Transudates often appear homogenic and anechoic. Exudates are also often anechoic, but if the density of the pleural fluid increases, like in coagulated blood, pus or chylothorax, then a more hypoechoic appearance is evident. Hyperechoic particles (also known as “plankton” or “the swirling sign”) can sometimes be seen floating around in the effusion [7, 194, 198, 200]. This finding is almost exclusively seen in exudates and in one study was shown to be a marker of a malignant pleural effusion [200]. In the case of empyema or complicated parapneumonic effusion, septa within the fluid and possible pleural thickening can often be seen using LUS [201]. Both septations and thickening of the pleura can however also be seen in malignancy and chronic pleural effusions [195, 202]. The presence of septations within a pleural effusion can have a high degree of impact in the choice of treatment and drainage. LUS is superior to CT for the detection of septations and hence is a key imaging modality in the assessment and subsequent treatment of patients with pleural effusion [203, 115]

#### Obstructive pulmonary disease

In the vast majorities of cases, patients with chronic obstructive pulmonary disease (COPD) or asthma both have normal LUS findings during exacerbations [107, 108, 110, 112, 204]. Even though LUS cannot diagnose obstructive pulmonary diseases, it has a key role in assessing these patients since LUS can rule-out both complications and associated diseases [107, 108, 110, 112, 204].

#### LUS used in other settings

Many studies of LUS in the pre-hospital setting exist [204–206] and display similar ability to diagnose IS, confirm or negate pulmonary oedema and verify endotracheal tube placement [204–206]. The ease of which portable ultrasound machines can be carried almost anywhere allows LUS to be used as a unique assessment tool in respiratory emergencies both at sea, mountainous terrain and even in outer space [114, 116, 207–209].

#### LUS-guided procedures

Most current guidelines recommend that, whenever possible, thoracocentesis should be performed under US guidance [210], as the number of complications is markedly reduced compared with conventional thoracocentesis. US-guided trans-thoracic biopsy of peripheral pulmonary lesions is now routinely employed [211–232]. So is LUS for ruling out pneumothorax after invasive procedures such as biopsy, drainage or placement of central venous catheters [233–238]. In trauma patients with pneumothorax, LUS quantification of the size of the pneumothorax can be used to predict whether removal of the chest tube can be performed safely or whether chest drainage should continue [106]. As an adjunct before thoracic surgery, LUS of the areas where the surgeon is expected to make an incision can diagnose and rule out pleural adhesions. If lung sliding is present, there are no pleural adhesions in the intended surgical site. If no lung sliding is present, adhesions should be suspected and the incision should be performed elsewhere [239]. Lung recruitment following positive end-expiratory pressure can be assessed with LUS [158, 178]. In a small study, LUS quantification of aeration changes during a trial of spontaneous breathing seemed to be able to predict post-extubation distress [240].

#### Extended focused assessment with sonography for trauma (EFAST)

FAST (focused assessment with sonography for trauma) is a simple, quick and safe method for the diagnosis of free fluid in the pericardium and peritoneum of trauma patients [241]. Many studies have demonstrated that LUS performs better than conventional chest X-ray in the diagnosis of pneumothorax and haemothorax in trauma patients [96–100, 177, 242, 243], prompting the implementation of EFAST with additional LUS for faster and better diagnosis of pneumothorax and haemothorax. One should follow the ABCD (airway, breathing, circulation and disability) principles when performing EFAST and start with LUS (B principle) followed by FAST (C principle). In order to save time, it is advantageous to only utilise the low-frequency abdominal transducer when performing EFAST, as the high-frequency transducer only has a penetration of a maximum 2–5 cm and cannot be used for the abdominal US examination. FAST should preferably be performed in less than 60 s. EFAST should be implemented at a maximum of 90–120 s. Like any other paraclinical examination, EFAST is just a snapshot and it is important to constantly assess the patient’s clinical condition, and repeat the EFAST should changes occur. With modern, advanced and mobile equipment, EFAST can be performed in the pre-hospital situation. This is advantageous as it not only allows for appropriate initiation of pre-hospital treatment and appropriate receiving facility, it provides the receiving trauma centre with essential information that will influence decisions on how best to transport the patient, e.g. via ambulance or helicopter, and buys the trauma facility precious time to prepare for the arrival of the trauma patient and plan essential treatment, e.g. acute surgery [244].

#### Confirmation of gastric tube placement

Abdominal ultrasonography performed in the intensive care unit had a 97 % sensitivity for detecting correct gastric placement of a weighted-tip nasogastric tube (NGT) in a mean (median, range) 24 min (11, 53) compared with radiography (100 % sensitivity) that took considerably longer, 180 min (113, 240). Hence, bedside sonography is a sensitive method for confirming NGT position, and can be easily taught to ICU physicians. Conventional radiography should be reserved for sonographically inconclusive cases [245]. Pre-hospital insertion of gastric tubes for gastric decompression was also successfully confirmed with ultrasonography by emergency physicians. [246]

A Sengstaken-Blakemore tube applied for severe oesophageal variceal bleeding has had considerable complications, including deaths, reported from oesophageal rupture after inadvertent inflation of the gastric balloon in the oesophagus [247]. Ultrasonography of the stomach can aid in the rapid confirmation of correct placement. If the Sengstaken tube is not directly visible, inflation of 50 ml air via the stomach (not the gastric balloon) lumen of the tube should lead to a characteristic jet of echogenic bubbles within the stomach. The gastric balloon is slowly inflated under direct sonographic control and usually appears as a growing echogenic circle within the stomach [247].

#### Prediction of successful extubation

Four adult ventilated patients who developed post-extubation stridor were found to have a significantly narrower column of air compared with the patients who did not develop stridor when the ultrasound transducer was placed on the cricothyroid membrane with a transverse view of the larynx [248]; these results need to be evaluated in larger studies. Intubated patients receiving mechanical ventilation in a medical intensive care unit had their breathing force evaluated by US. The probe was placed along the right anterior axillary line and the left posterior axillary line for measurement of liver and spleen displacement in cranio-caudal aspects, respectively. The cut-off value of diaphragmatic displacement for predicting successful extubation was determined to be 1.1 cm. The measured liver and spleen displacements are thought to reflect the “global” functions of the respiratory muscles and this method is purported to be a good parameter of respiratory muscle endurance and predictor of extubation success [35].

#### LUS-guided management of acute breathlessness during pregnancy

The usage of LUS is attractive in pregnancy as it does not use ionising radiation. Its safety and non-invasiveness is a vital resource in diagnosing acute respiratory failure in patients. Dyspnoea in pregnancy can be due to non-cardiogenic pulmonary oedema secondary to asthma, embolic disorders, pneumonia, exacerbation of underlying disease or cardiogenic pulmonary oedema. LUS has been shown to aid management of lung disorders in pregnant women, by allowing differentiation from a presumed asthmatic exacerbation when vertical B-lines were seen, indicating severe pulmonary oedema. Detection of interstitial oedema at a pre-clinical stage also allowed adequate fluid resuscitation in a pre-eclamptic patient at high risk of alveolar pulmonary oedema [249].

#### Special techniques and indications and future aspects

The lateral position of a laryngeal mask airway cuff can be seen if the cuff is filled with fluid but the fluid damages the cuff on subsequent autoclaving [73]. Airway obstruction due to a prevertebral haematoma following difficult central line insertion may be prevented by using US for this procedure [250]. Endoscopic high-frequency ultrasound of the larynx has been described: the larynx is depicted from the luminal side by filling the larynx and the trachea above the cuff of the endotracheal tube with 0.9 % saline to obtain sufficient tissue connection and prevent the retention of air bubbles in the anterior commissure [251]. The technique involves a thin catheter high frequency probe with a rotating mirror to spread the ultrasound ray, producing a 360° image rectilinear to the catheter [251, 252]. Three-dimensional and pocket-sized US devices are likely to move the boundaries for both the quality and the availability of ultrasonographic imaging of the airway. Even a pocket-sized smartphone-based system allows ultrasonography in a quality acceptable for airway evaluation [253].

#### Education: how to learn airway US

The following studies give an insight into what requirements, and how little, it takes to learn basic airway US. After 8.5 h of focused training (2.5-h didactic course covering essential views of normal and pathological conditions; and three hands-on sessions of 2 h duration), physicians without previous knowledge of US could competently perform basic general ultrasonic examinations [254]. The examinations were aimed at diagnosing the presence of pleural effusion, intra-abdominal effusion, acute cholecystitis, intrahepatic biliary duct dilation, obstructive uropathy, chronic renal disease, and deep venous thrombosis. For questions with a potential therapeutic impact, the physicians answered 95 % of the questions correctly [254]. The acquisition of sonographic skills to make a correct diagnosis is more task specific, meaning, the basic skill required to detect a pleural effusion may be acquired in minutes and may then improve with experience [255]. In a cadaver model where a 7.5-MHz curved transducer was placed longitudinally over the cricothyroid membrane, it was possible for residents given only 5 min of training in the technique to correctly identify oesophageal intubation (97 % sensitivity) when this was performed dynamically, during the intubation. When the examination was performed after the intubation, the sensitivity was poor [62]. A 25-min instructional session, including both a didactic portion and hands-on practice, was given to critical care paramedics/nurses who were part of a helicopter critical care transport team. The instructional session focused solely on the detection of the presence or absence of lung sliding, involving secondary ultrasonographic techniques for the detection of lung sliding, including power Doppler and M-mode US. The participant’s performance was studied on fresh cadavers. The presence or absence of the lung sliding was correctly identified in 46 of the 48 trials, for a sensitivity and specificity of 96.9 % and 93.8 %. At a 9-month follow-up, the presence or absence of the lung sliding was correctly identified in all 56 trials, resulting in a sensitivity and specificity of 100 % [256]. In a study of whether a 2-h training course would allow emergency physicians to accurately diagnose post-traumatic pneumothorax, LUS performed by the course trainees had a sensitivity of 86.4 %, a specificity of 100 %, a positive predictive value of 100 % and a negative predictive value of 95.6 % [257]. An evaluation of a 2-h course with didactic lessons on LUS and diagnostic criteria for pneumothorax and pulmonary oedema showed that pre-hospital physicians (with varying experience with ultrasound) improved significantly from their pre- to post-course test [258]. Physicians with no experience in LUS, apart from a 30-min lecture on how to diagnose B-lines using LUS was compared with LUS-experienced physicians. Twenty consecutive patients were assessed for B-lines both by one of the experienced physicians, using a “high-tech” ultrasound apparatus and one of the inexperienced physicians using a “low-tech” hand-held portable ultrasound apparatus. For the assessment of B-lines, there was a tight correlation (*r* = 0.958) between the two observations in the same patient by “high-tech veteran” and “low-tech beginner” [259]. The simplicity of LUS and its potential for more widespread use by inexperienced operators devoid of ultrasound experience have been illustrated in a study where telementoring by an expert in LUS enabled a person with no prior experience in ultrasonography to diagnose and exclude apnoea and pneumothorax [260]. The above studies show a rapid learning curve and this is of benefit in trying to train a suitably sized workforce. There is a need for ongoing and additional training to maintain the basic ultrasonography curriculum. Objective Structured Assessment of Ultrasound Skills (OSAUS) is a framework designed for in-training assessment of ultrasound competence. Newly published studies conclude that ultrasound competence can be assessed in a reliable and valid way using the OSAUS scale. The pass/fail-scores may be used to help determine when trainees are qualified for independent practice [261]. The numbers of supervised ultrasound examinations required to maintain competence is debatable. Our recommendation is a minimum of 25 supervised ultrasonography for each procedure, i.e. ultrasound-guided intubation or diagnostic lung ultrasonography. Further research regarding this subject is warranted.

During US training, acknowledgement of the intrinsic drawbacks of ultrasound and the minimisation of their impact on clinical service should be made. Ultrasound will always be operator dependent and reproducibility of findings is an essential part of US training.

## Conclusions

- US has many advantages for imaging the airway—it is safe, quick, repeatable, portable, widely available and gives real-time dynamic images relevant for several aspects of management of the airway.
- US must be used *dynamically* for maximum benefit in airway management and in direct conjunction with the airway management: immediately before, during and after, airway interventions.
- US can be used for direct observation of whether the tube enters the trachea or the oesophagus by placing the ultrasound probe transversely on the neck at the level of the suprasternal notch during intubation, thus confirming intubation *without* the need for ventilation or circulation.
- US can be applied before anaesthesia induction and can diagnose several conditions that affect airway management, e.g. caution in inserting laryngeal masks when sialolithiasis is present, diagnosing obstructive sleep apnoea and evaluating the nature of stomach contents
- US can identify the croicothyroid membrane prior to management of a difficult airway, thus preparing for a possible emergency cricothyroidotomy.
- US should be the first diagnostic approach when a pneumothorax is suspected intraoperatively, during initial trauma evaluation or following invasive procedures related to the airways.
- US can rival fibre-optic examination for confirmation of the correct placement of an LMA in assessing adequacy of the laryngeal seal and pulmonary ventilation.
- US can predict the appropriate diameter of endotracheal, endobronchial or tracheostomy tube size:
- US can guide airway-related nerve blocks by potentially identifying the superior laryngeal nerve space if not the nerve itself, for more accurate deposition of local anaesthetic.
- US can confirm ventilation by observing lung sliding bilaterally.
- US can improve percutaneous dilatational tracheostomy by identifying the correct tracheal-ring interspace, avoiding blood vessels and determining the depth from the skin to the tracheal wall.
- US can with high sensitivity and specificity diagnose pneumothorax, interstitial syndrome (e.g. pulmonary oedema), lung consolidations (e.g. pneumonia, pulmonary embolism), atelectasis and pleural effusion in patients with acute dyspnoea.
- US can improve patient safety by performing procedures under US guidance, e.g. thoracocentesis, vascular line access, guide timing of removal of chest tubes by quantification of residual pneumothorax size.
- US can extend the role of sonographic assessment of trauma victims by including LUS assessment for pneumothorax and haemothorax in the FAST protocol
- US can confirm gastric tube placement
- US may be limited due to a variety of factors such as patient habitus (i.e. bariatric patients). Therefore in some cases it still may be necessary to supplement ultrasonography with other imaging modalities.

With the increasing availability of portable bedside ultrasound machines that are not only functional but extremely user-friendly, the skill of US in airway management should be embraced by all clinicians for all the reasons stipulated above.

## Abbreviations

ABCD: Airway, breathing, circulation and disability
CM: Cricothyroid membrane
CT: Computed tomography
EBUS: Endobronchial ultrasound
EFAST: Extended focused assessment with sonography for trauma
FAST: Focused assessment with sonography for trauma
ICU: Intensive care unit
IS: Interstitial syndrome
LMA: Laryngeal mask airway
LUS: Lung ultrasound
NGT: Nasogastric tube
OSA: Obstructive sleep apnea (OSA)
PDT: Percutaneous dilatational tracheostomy
US: Ultrasonography
